# Mobile eHealth Interventions for Obesity: A Timely Opportunity to Leverage Convergence Trends

**DOI:** 10.2196/jmir.7.5.e58

**Published:** 2005-12-20

**Authors:** James T Tufano, Bryant T Karras

**Affiliations:** ^2^Department of Health ServicesUniversity of WashingtonSchool of Public Health and Community MedicineSeattle WAUSA; ^1^Department of Medical Education and Biomedical InformaticsUniversity of Washington School of MedicineSeattleWAUSA

**Keywords:** eHealth, obesity, intervention, mobile computing, cellular telephone, weight loss, health behavior, health communication, behavior modification, consumer health informatics

## Abstract

Obesity is often cited as the most prevalent chronic health condition and highest priority public health problem in the United States. There is a limited but growing body of evidence suggesting that mobile eHealth behavioral interventions, if properly designed, may be effective in promoting and sustaining successful weight loss and weight maintenance behavior changes.

This paper reviews the current literature on the successes and failures of public health, provider-administered, and self-managed behavioral health interventions for weight loss. The prevailing theories of health behavior change are discussed from the perspective of how this knowledge can serve as an evidence base to inform the design of mobile eHealth weight loss interventions. Tailored informational interventions, which, in recent years, have proven to be the most effective form of conventional health behavior intervention for weight loss, are discussed. Lessons learned from the success of conventional tailored informational interventions and the early successes of desktop computer–assisted self-help weight management interventions are presented, as are design principles suggested by Social Cognitive Theory and the Social Marketing Model.

Relevant computing and communications technology convergence trends are also discussed. The recent trends in rapid advancement, convergence, and public adoption of Web-enabled cellular telephone and wireless personal digital assistant (PDA) devices provide timely opportunities to deliver the mass customization capabilities, reach, and interactivity required for the development, administration, and adoption of effective population-level eHealth tailored informational interventions for obesity.

## Introduction

Obesity is often cited as the most prevalent chronic health condition and highest priority public health problem in the United States, and it has also gained recognition as a global public health concern. The increasing prevalence of obesity-related diseases such as type 2 diabetes and cardiovascular disease has lead the medical and public health communities to declare an obesity epidemic and to conclude that more effective population-oriented interventions are needed [1].

There is a limited but growing body of evidence suggesting that eHealth interventions exhibit potential to address a variety of chronic illnesses that can be effectively treated via behavior modification [2]. Also, it appears that there is a significant and growing opportunity for eHealth obesity intervention designers to leverage the widespread public adoption of rapidly converging information and communication technologies—most notably the World Wide Web, wireless PDAs, and cellular telephones.

### The Failures of Conventional Behavioral Interventions

An Institute of Medicine report published in 2000 concluded that “behavioral and social interventions offer great promise to reduce disease morbidity and mortality, but as yet their potential to improve the public’s health has been relatively poorly tapped” [3].

This claim is further validated by trends exhibited in US public health data collected via the Behavioral Risk Factor Surveillance System (BRFSS) program of the Centers for Disease Control and Prevention (CDC). The BRFSS is a cross-sectional telephone survey program administered by the CDC in conjunction with state health departments. Data collected from each state are pooled to produce nationally representative estimates of the prevalence of specific health behaviors that affect risk for one or more of the ten leading causes of death in the United States. According to the results of the 2000 BRFSS, which are based on responses from 184450 participants in 50 states, the prevalence of obesity was 19.8% among US adults, representing a 61% increase compared to the 1991 BRFSS data. Also, 56.4% of 2000 BRFSS survey respondents were overweight (body mass index ≥ 25 kg/m^2^) compared to 45% in 1991 [4].

The 2000 BRFSS data also showed that 38.5% of US adults reported that they were attempting to lose weight, compared to 36.6% in 1991; 35.9% were trying to maintain weight, compared to 34.4% in 1991; and 25.6% were doing neither, compared to 29.0% in 1991 [5]. This would indicate that Americans are collectively putting more effort into losing or maintaining weight, or at least *perceive* that they are putting more effort into weight reduction or maintenance, yet as a whole are exhibiting decreasing levels of success.

#### Provider-Delivered Health Behavior Interventions

Behavior changes related to dietary intake and physical activity might be effective in preventing and treating obesity. Studies have shown that overweight and obese people are more likely to attempt weight loss by adopting lifestyle changes if they are advised to do so by a health care professional [6], yet the 2000 BFRSS data showed that only 42.8% of the obese respondents who had received a routine medical checkup in the past year had been advised to lose weight by their providers [4].

An explanation is needed for why 57.2% of American clinicians are failing to advise their obese patients to lose weight during routine medical checkups. This clinical window of opportunity for engaging patients in discussion about obesity and their health is being missed. Various explanations are offered in the literature, including lack of provider access to high-quality information about effective patient intervention strategies, lack of access to appropriate support services, and a lack of provider motivation to work with obese and overweight patients [7]. A compelling explanation is that the provider community is skeptical of the efficacy of conventional provider-delivered health behavior change interventions—an explanation supported by a systematic review of the existing literature on provider-delivered and health care organization–delivered behavioral health interventions that was conducted in 2002 [8]. In this review, Harvey et al concluded that relatively few obesity interventions have been evaluated rigorously, and “at present, there are few solid leads about improving obesity management.” The recent systematic reviews of the literature on dietary and physical activity counseling, used to inform the recommendations of the US Preventive Services Task Force, have yielded similar results [9].

#### Conventional Population-Oriented Public Health Interventions

Health communication campaigns provide the traditional means by which public health organizations have sought to promote health behavior change. Conventional health communication campaigns involve dissemination of messages from experts to the public through mass media channels, with the intent of motivating the public to adopt specific behaviors that have been proven to reduce the risk of disease. However, there is evidence that traditional health communication interventions exhibit a high rate of failure to promote behavior change [10] and that traditional methods of health communication are particularly ineffective at addressing weight-related health behavior change [11].

Two examples of the failure of public health communication interventions aimed at obesity are the California Five-A-Day for Better Health Campaign and the former Health Education Authority (HEA) of England’s Active for Life campaign. The California Five-A-Day Campaign was an intensive five-year statewide intervention aimed at promoting dietary behavior change by increasing the consumption of fresh fruits and vegetables. Program evaluation showed that knowledge and understanding of the importance of eating more fruits and vegetables increased substantially among Californians as a result, but this did not result in any measurable behavior change. There was no increase in fruit and vegetable consumption in any population group, and among Hispanic adults, consumption actually dropped by 18% [12].

The largest-scale public health intervention attempted in Europe during the latter part of the 1990s was the Active for Life campaign, a three-year intervention that aimed to promote moderate-intensity physical activity as a part of everyday life. The multi-faceted intervention ran from 1996 to 1999 and included a variety of mass media communication components, as well as a program of support to health care and public health professionals who worked to develop and promote localized community-based physical activity programs. Evaluation showed that, after three years, there was no evidence that the campaign improved physical activity, either overall or in any subgroup [13].

These studies highlight the need for the public health practitioners and policy makers to explore alternative methods of promoting health behavior change in the fight against the obesity epidemic.

### Prevailing Theories of Health Behavior Change and Intervention Models

Prevailing theories of health behavior change suggest several root causes for the failure of conventional obesity interventions. These theories and their supporting empirics also imply key intervention design features that may increase their likelihood of promoting and sustaining the desired behavior changes.

Several established theories and models of health-related behavior have informed the design of successful health behavior change communication interventions. These theories and models are drawn mostly from the fields of psychology, sociology, communication, and medicine, and they draw heavily on research in persuasion, social marketing, and relational communication [14]. The most prevalent health behavior theories cited by intervention designers include the Health Belief Model, the Theory of Reasoned Action, the Transtheoretical (or Stages of Change) Model, Learning and Conditioning Theory, Decision-Making Theory, and the Diffusion of Innovations Model. These various theories and models share the common objective of attempting to explain and predict individual health-related behaviors, and they have formed the basis for the design, deployment, and evaluation of the majority of health behavior interventions at the individual, organizational/group, and population levels [15].

Of the dominant classic models, the Transtheoretical Model and the Health Belief Model have been drawn on most heavily in the design of tailored informational interventions, which, in recent years, have generally proven to be the most effective model of health behavior change intervention for a variety of diseases [16,17]. In their 2002 systematic review, Ryan and Lauver suggest that tailored informational interventions exhibit four key defining characteristics that differentiate them from other intervention types [16]. According to their definition, tailored informational interventions include (1) an assessment of key characteristics of each targeted person; (2) small units of content prepared to match each of these key characteristics, often stored in a message library; (3) a decision algorithm that provides the matching logic; and (4) a designated information delivery channel or mechanism (eg, print, email, telephone) [16].

Tailored informational interventions are based on the premise that the design of a behavioral communication intervention must be modified or “tailored” to accommodate varying states of readiness for behavior change among the targeted recipients. Under this design paradigm, delivering the right message at the right time often requires both multiple forms of communication delivery and variation in the content of messages delivered. They are typically used to support individual provider-to-patient intervention strategies and small-scale health promotion programs, often involve the use of computer-generated content construction, and are gaining broad acceptance as one of the only forms of health communication intervention that successfully yields behavior change [16-18].

The Transtheoretical (or Stages of Change) Model is based on the premise that people move across a continuum of readiness to change, moving from “pre-contemplation” (e.g., “I suppose that I *should* try to eat healthier and shed some pounds at some point…”) to “action” (e.g., “Today I am purging my fridge and cupboard of all junk food…”) to “maintenance” of a behavior change (e.g., “I have been planning my meals and buying only healthy foods for the past eight months…”)of a behavior change. Tailored informational interventions leverage the Transtheoretical Model by designing incorporating communication mechanisms and content specific to a targeted individual’s state of readiness for change as indicated by his or her position on this continuum [19].

The Health Belief Model estimates a person’s likelihood of adopting a healthy behavior based on his or her perceptions of the risk of becoming ill, anticipated benefits to be gained, and the barriers to adoption of the behavior change. Tailored informational interventions leverage this model primarily in the design of the content of the messages delivered [20].

These and most of the other classic theories and models of health behavior change emphasize the individual as the decision maker, which drives intervention designers to focus on delivering expert-driven, risk-based information to targeted at-risk individuals. Targeted individuals are expected to use this information to make rational decisions about discrete behavior change and then act on these rational decisions by changing their behaviors. Emmons cites this as a fundamental weakness and argues that improving the effectiveness of health behavior interventions will require models of behavior that account for how mediating variables of behavior change are influenced by sociocultural dynamics [21]. Growing agreement within the public health community has lead to exploration of interventional approaches that leverage Social Marketing Theory, Social Cognitive Theory, and other behavioral science and social epidemiology theories that place a greater emphasis on the social, institutional, and cultural contexts that impact an individual’s behaviors [22].

Social Cognitive Theory shares some attributes of both the Transtheoretical Model and the Health Belief Model, but it offers some unique and compelling contributions in its emphasis on the role of personal empowerment in behavior change. This theory suggests that individuals’ sense of “self-efficacy” or agency about a behavior and their perceived ability to cope with and control situations are core determinants of behavior change [23].

## Successful Health Behavior Interventions for Obesity

In assessing the literature on behavioral obesity interventions, careful consideration must be given to the magnitude and duration of the primary outcomes. Many “successful” obesity interventions produce significant weight loss that is difficult to maintain or result in weight losses that are too small to yield a substantial health gain. There is a paucity of compelling evidence that any behavioral obesity interventions consistently yield both clinically significant and sustainable weight loss. Given this caveat, several studies nonetheless provide encouraging insights into intervention characteristics that, if considered in the design of eHealth obesity interventions, may improve their likelihood of success.

In their systematic review of studies of health behavior interventions aimed at increasing physical activity published from 1983 to 1997, Marcus et al identified a total of 28 qualifying papers [11]. Seven described studies of traditional mass media health communication campaigns conducted at the state or national level. None of these interventions were found to have affected behavior change. However, in this same review, the majority of the other 21 studies were found to have had some positive impact on exercise-related behaviors. They were all smaller-scale interventions using various forms and combinations of print and/or telephone media. The majority of these interventions exhibited design characteristics based heavily on the Transtheoretical Model and/or Social Cognitive Theory, and most could be classified as tailored informational interventions. Three of these studies reported that adherence rates were better for home-based exercise programs augmented with telephone contacts than for structured programs entailing face-to-face contact.

The findings of two systematic reviews conducted in 2002 and 2004 formed the foundation for physical activity intervention recommendations offered by the CDC-sponsored Guide to Community Preventive Services [24]. Both stressed the importance of tailoring to individual and/or targeted population characteristics.

Marcus and Heimendinger’s randomized controlled trial of a tailored informational intervention targeting dietary behavior change demonstrated its effectiveness in improving eating behaviors that significantly improved fruit and vegetable consumption [25]. Further evidence of the promise offered by tailored informational interventions that incorporate information technologies is evident in Brug et al’s 1999 review of the literature. The authors concluded that computer-tailored nutrition education is more likely to be read, remembered, and experienced as personally relevant compared to standard materials. They also found that interventions incorporating computer-generated personalized nutrition education delivered via tailored informational interventions are effective in promoting desired dietary behavior changes [18]. Similar results were published in 2001 by Bull et al, who found that tailored health education materials were significantly more effective than nontailored materials at changing dietary behaviors associated with weight loss interventions [26].

O’Neil concluded that self-monitoring on an ongoing basis is a key component of effective dietary behavior change, that self-monitoring enhances weight loss outcomes, and that information technology advances offer promise in improving compliance and effectiveness of self-monitoring [27]. Similarly, in their 2002 systematic review of tailored informational intervention outcomes studies, Ryan et al concluded that they are more effective when ipsative feedback (eg, comparing current to past behavior) was included as a feature of the intervention [16]. Brug et al reached similar conclusions in their 1998 study of the impact of computer-tailored iterative feedback on fat, fruit, and vegetable consumption [28].

Further encouraging findings for eHealth interventions are offered in Latner’s 2001 review of obesity self-treatment interventions that included studies of computer-assisted obesity interventions [29]. Several interventions were identified that described pilot intervention studies demonstrating the effectiveness of computer-assisted self-monitoring of food intake and exercise, goal setting, response-contingent feedback, and regular auditory prompts reminding users to enter self-reports. One study was identified that showed no significant difference between the outcomes of computer-assisted self-therapy and a conventional weight loss program using therapist-conducted treatment. Although weight loss was modest in both groups, this study may indicate the potential for substitution of computer-assisted, self-therapy weight loss interventions for more costly and inconvenient provider-delivered interventions.

Of particular interest to eHealth intervention designers working with cellular telephone platforms are Kreps et al’s conclusions that conventional (land-line) telephone-delivered tailored informational interventions are generally more effective at promoting health behavior change than printed media interventions [30]. Other studies have shown that tailored informational interventions that utilize combinations of print and telephone-delivered interventions can be highly effective [31].

Also worth noting is the recent trend among commercial weight loss programs such as Weight Watchers and Jenny Craig to incorporate Web-based, self-help tools into their programs [32,33]. Although there are no evaluation studies of these tools currently available in the published literature, there is limited evidence that these commercial weight loss programs in their totality may be effective in promoting behavior changes that yield sustained weight loss among their enrollees [34].

### Lessons Learned for eHealth Intervention Development

The potential to leverage eHealth behavioral interventions to improve weight management behaviors appears to be significant. The successes of both computer-assisted, self-help interventions for obesity and tailored informational interventions for a variety of health conditions provide a limited but valuable evidence base that can be leveraged to inform the design of effective eHealth obesity interventions.

Revere and Dunbar’s 2000 systematic review of computer-generated outpatient health behavior intervention studies included 37 eligible studies that were published from 1996 to 1999. Of these 37 clinical trials, 34 (91.9%) reported either statistically significant or improved outcomes, and 23 were classified as exhibiting the tailored informational intervention design [2]. These findings would imply that the tenets of conventional tailored informational intervention design translate well to the design of eHealth interventions.

Neuhauser and Kreps conducted an extensive review of the literature on health behavior theory and conventional health behavior intervention outcomes studies in 2003 [15]. Not surprisingly, they found that tailored communication is more effective than generic messages in promoting health behavior change and that health communication is more effective when it reaches people on an emotional as well as a rational level. They cite both social influence theory (eg, Social Cognitive Theory, Social Marketing Model) and evidence from intervention outcomes studies to build a compelling case that interactivity may be the most important trait of effective health behavior interventions and that the involvement of targeted recipients in the design and engagement of the health communication intervention increases the likelihood that they will adopt the desired behaviors. They also suggest that a combination of the effectiveness of interpersonal communication and the reach of mass media communication is needed to change population behavior. This conclusion would appear to have significant implications for eHealth intervention designers working with cell phone and voice over Internet protocol (VoIP) technologies.

The concept of active construction of information bundles, while not an established theory of health behavior change, is also worthy of consideration. Gorman et al’s 2001 paper [35] discusses the relationships between individuals’ information management activities and maintenance of situation awareness. They assert that as people actively and consciously engage in the creation and/or organization of information into “bundles” to support specific tasks, the act of actively processing and manipulating information improves their understanding and situational awareness. They also cite additional studies [36,37] that support the notion that “over automation” of data entry and information processing may diminish both the users’ situation awareness and the usefulness of the information, lowering the probability of achieving the desired outcomes. Applied to eHealth intervention design, this concept would suggest that some data entry and application configuration tasks that could be automated should instead remain in the foreground and fully visible to the end user. The acts of consciously entering and organizing data (eg, estimating food portions, calculating and entering nutritional values for foods eaten) may be just as (or more?) relevant to promoting the desired behavior changes than retrospective use of the captured data.

### Technology Adoption: Crossing the Chasm or Finding the Bridges?

As previously discussed, both conventional and eHealth tailored informational interventions have proven to be successful for a variety of health conditions when delivered as individual or small-scale, provider-to-patient interventions. But the difficulties of individual tailoring of message content and interactive delivery have hindered their application to the design of large-scale public health interventions. However, the trend toward rapid convergence of these technologies [38,39] enables a wide variety of desirable eHealth intervention design features that were previously not feasible. Three of the most significant features are interactivity, self-configuration and customization, and mass customization of organizationally sponsored informational interventions (eg, administered by public health organizations, health care providers, health maintenance organizations, or commercial weight loss programs).

Cell phones and networked PDAs enable interactive voice and text communication. However, convergence trends have added real- and near-time multimedia communication capabilities to both. As these device technologies converge, voice-, text-, and multimedia communication modalities are supported in a unified device. Furthermore, as the computing power and memory of these devices increases, users are becoming more empowered to self-configure applications. The content and timing of alerts, reminders, and text memos can be easily customized, and users are increasingly enabled to customize the look, feel, and organization of the user interface of applications running on their cell phones, PDAs, and desktop computers. This technology convergence is an opportunity to deliver organizationally sponsored eHealth obesity interventions.

Health behavior interventions must *reach* the public in order to succeed in promoting and maintaining health behavior change at the population level. eHealth behavioral interventions must therefore be designed and deployed using existing technology development and adoption trends rather than introducing new devices/technologies. By this line of reasoning, the widespread public adoption of cellular telephones [40], wireless PDAs, and use of the Web represent pervasive and rapidly expanding and converging technology adoption trends that should be leveraged in any population-oriented eHealth obesity intervention aiming to reach beyond the desktop. Also, the same newly enabled features of interactivity and self-configuration also provide public health officials with easy facilities for developing and administering flexible and tailored interventions to better meet the needs of specific targeted populations. The trend toward information technology–enabled mass customization of service design and delivery that revolutionized other industries during the 1990s [41] is now possible in public health, medicine, and the commercial weight loss industry.

Table 1 demonstrates, through scenarios, how an eHealth obesity intervention designer might employ the theories, empirical evidence, and technology convergence trends we have discussed.

**Table 1:** Scenarios illustrating how an eHealth obesity intervention designer might employ the theories, empirical evidence, and technology convergence trends discussed in this article

**Convergence-Enabled Feature**	**Example Use Case**
Interactivity	Before eating a meal, a user borrows her friend’s Blackberry to access her Web diet journal. She checks the remaining balance in her daily calorie budget, enters the number of calories she wishes to “spend” on the given meal, and is then presented with a personalized list of her “favorite healthy foods” that fall within the range.
Self-Configuration and Customization	A user creates an alert to text message himself at 11:45 am daily with the message “drink your water before going to lunch.”
Mass Customization	Weight Watchers clients enroll in a service that sends reminders and Web forms to their smart phones. When opened, they are automatically localized to the recipients’ language, food preferences, and FlexPoints targets based on their unique configuration settings.

## Conclusions

One study published in 2004 estimated that by the end of 2003, over 60% of all US adults owned an activated cellular telephone, with this statistic growing at slightly over 5% annually [42]. Thus, it would appear that cell phones would be the preferred hardware platform for eHealth obesity interventions for reasons of both enabling effective intervention design features and for promoting rapid public adoption and acceptance.

In their 2000 systematic review of mobile eHealth intervention studies, Revere and Dunbar concluded that “future studies need to identify which [eHealth intervention] models are best suited to which health behavior, whether certain delivery devices are more appropriate for different health behaviors, and how care can benefit from patients’ use of portable devices” [2]. We conclude that the appropriate model for obesity and weight management is the tailored informational intervention modified according to design principles suggested by Social Cognitive Theory and the Social Marketing Model. The health behaviors to target are self-monitoring of diet and physical activity. The devices are Web-enabled “smart” cellular telephones and wireless PDAs. Given the lack of effectiveness of other interventions to prevent or treat obesity in a sustainable matter, trials of these persuasive, ubiquitous technologies are required without delay.

## Abbreviations

BRFSS: Behavioral Risk Factor Surveillance System
CDC: Centers for Disease Control and Prevention
PDA: personal digital assistant
VoIP: voice over Internet protocol
